# Long-term effect of coffee consumption on autosomal dominant polycystic kidneys disease progression: results from the Suisse ADPKD, a Prospective Longitudinal Cohort Study

**DOI:** 10.1007/s40620-017-0396-8

**Published:** 2017-04-06

**Authors:** Laura Girardat-Rotar, Milo A. Puhan, Julia Braun, Andreas L. Serra

**Affiliations:** 1 Epidemiology, Biostatistics and Prevention Institute, University of Zurich, Hirschengraben 84, 8001 Zurich, Switzerland; 2 Department of Internal Medicine and Nephrology, Medizinisches Kompetenzzentrum für ADPKD, Suisse ADPKD, Hirslanden, Zurich, Switzerland

**Keywords:** ADPKD, Coffee, Epidemiology, Kidney volume, Renal function decline, Progression

## Abstract

**Background:**

Previous in vitro experiments of human polycystic kidney disease (PKD) cells reported that caffeine is a risk factor for the promotion of cyst enlargement in patients with autosomal dominant PKD (ADPKD). The relentless progression of ADPKD inclines the majority of physicians to advocate minimization of caffeine consumption despite the absence of clinical data supporting such a recommendation so far. This is the first clinical study to assess prospectively the association between coffee consumption and disease progression in a longitudinal ADPKD cohort.

**Methods:**

Information on coffee consumption and disease progression was collected at each follow-up visit using standardized measurement methods. The main model for the outcomes, kidney size (height-adjusted total kidney volume, htTKV) and kidney function (estimated glomerular filtration rate, eGFR), was a linear mixed model. Patients entered the on-going Swiss ADPKD study between 2006 and June 2014 and had at least 1 visit every year. The sample size of the study population was 151 with a median follow-up of 4 visits per patient and a median follow-up time of 4.38 years.

**Results:**

After multivariate adjustment for age, smoking, hypertension, sex, body mass index and an interaction term (coffee*visit), coffee drinkers did not have a statistically significantly different kidney size compared to non-coffee drinkers (difference of −33.03 cm^3^ height adjusted TKV, 95% confidence interval (CI) from −72.41 to 6.34, p = 0.10). After the same adjustment, there was no statistically significant difference in eGFR between coffee and non-coffee drinkers (2.03 ml/min/1.73 m^2^, 95% CI from −0.31 to 4.31, p = 0.089).

**Conclusion:**

Data derived from our prospective longitudinal study do not confirm that drinking coffee is a risk factor for ADPKD progression.

**Electronic supplementary material:**

The online version of this article (doi:10.1007/s40620-017-0396-8) contains supplementary material, which is available to authorized users.

## Introduction

Belibi and colleagues reported in 2002 results derived from in vitro experiments of human polycystic kidney disease (PKD) cells exposed to caffeine [1]. Caffeine activated pro-proliferative signalling pathways and increased transepithelial fluid secretion in murine PKD cells and the authors concluded that caffeine is a risk factor for the promotion of cyst enlargement in patients with autosomal dominant PKD (ADPKD) [2]. Caffeine raises intracellular adenosine 3′:5′-cyclic monophosphate (cAMP) levels by competitively and non-selectively inhibiting cyclic nucleotide phosphodiesterase. These groups of enzymes degrade the phosphodiester bond in the second messenger cAMP molecules. For more than 10 years, intensive research has revealed that increased levels of intracellular cAMP causes ADPKD progression by stimulating transepithelial secretion and proliferation, thus further fostering the persuasion that caffeine consumption accelerates ADPKD progression [3, 4]. Since then, ADPKD experts and ADPKD patient organizations have advocated to minimize caffeine intake.

However, there is little evidence on the effect of caffeine in patients with ADPKD and only cross-sectional studies are available so far. In 2012, the results of a cross-sectional study in ADPKD and healthy volunteers investigating the difference in caffeine intake and renal volume were reported [5]. Vendramini and colleagues did not identify an association between caffeine intake and kidney size, but the intake of caffeine was much lower among ADPKD patients than healthy volunteers. Possibly the observed caffeine consumption difference among ADPKD patients and healthy volunteers was due to the conviction in the ADPKD community that caffeine might be toxic for ADPKD patients, although these concerns were based solely on in vitro human cell experiments [6].

The relentless progression of ADPKD and the lack of effective therapies until recently has inclined the majority of physicians to advocate minimization of caffeine consumption despite the absence of clinical data supporting their advice. In fact, cohort studies in human beings repeatedly showed a beneficial effect of coffee consumption on various outcomes. Coffee consumption has been associated with decreased mortality in a meta-analysis of nearly 1 million subjects in 21 different independent studies [3, 7]. Coffee drinking’s beneficial effects have been identified for various diseases, including cardiovascular and kidney diseases [8–10].

Since the current evidence base is very weak, our aim was to assess the longitudinal association of coffee consumption and the progression of disease in patients affected by ADPKD.

## Methods

### Study design and participants

Patients were eligible for our analysis if they were enrolled from 2006 to 2014 in the Swiss ADPKD cohort and if they had not been treated with possible disease modifying drugs (e.g. sirolimus, everolimus, tolvaptan, and somatostatin analogues). Patients had a proven ADPKD diagnosis, were between 18 and 60 years old and had an estimated glomerular filtration rate (eGFR) >30 ml/min/1.73 m^2^ at enrolment. All patients provided written informed consent and the local ethics committee approved the study (EK-number 1178). Possible serious adverse events and adverse events were reported to the study investigator at least at every study visit. The enrolled patients had a minimum of 1 and a maximum of 8 follow-up visits (median 4) and a median follow-up time of 4.38 years (interquartile range IQR 2.16–6.1). At each study visit the medical history was obtained, including medication, complications related to ADPKD, and the daily consumption of caffeine [11]. We excluded 69 patients with less than 2 visits because our study focused on disease progression over time. This gave a total sample size of 151 patients with 687 observations.

### Progression of disease

Our primary outcome for progression of disease was kidney size by using height adjusted total kidney volume (htTKV) and the secondary outcome was the eGFR. The baseline visit and each follow-up visit included a measurement of kidney size and function, using a standardized protocol. The magnetic resonance imaging (MRI) acquisition consisted of breath-hold T1-weighted fast spoiled gradient echo sequence without fat suppression sequence (4 mm slice thicknesses) and trans-axial T2 weighted fast spin echo sequences. The total kidney volume was estimated by hand contouring of all MRI slices. The observer was blinded to previous measurements. Manual volume segmentation was done with the Advantage Workstation 4.4, GE Healthcare, Little Chalfont, UK [12]. At each study visit, serum creatinine was assessed with the use of the modified Jaffé method traceable to an isotope-dilution mass spectroscopy reference [13]. GFR was estimated by applying the chronic kidney disease-epidemiology (CKD-EPI) formula [14].

### Coffee consumption

Coffee consumption was assessed at each visit according to the following categories: never drink coffee; drink <1 cup of coffee per day, 1–2 cups of coffee per day, 2–4 cups of coffee per day, and >4 cups of coffee per day. For the analysis, coffee consumption was summarised as a binary variable: “no or <1 coffee a day” vs. “coffee drinkers”. If the information about coffee consumption was missing for a certain visit (number of missing values: 162), the last available information was used for the analysis. We also performed a sensitivity analysis without imputation of missing values.

### Potential confounders

We considered the following potential confounders that could bias the association of coffee consumption and progression of ADPKD: at each visit, anthropometric measurement and laboratory examinations were performed including height, weight and blood pressure measurement as well as various blood and urine tests. Body mass index (BMI) (kg/m^2^) was included as a continuous variable. Blood pressure was measured in sitting position, twice at an interval of 10 min, using an oscillometric blood pressure device (Boso-Medicus, Jungingen, Germany). The variable hypertension was defined as either systolic blood pressure above 140 mmHg, diastolic blood pressure above 90 mmHg and/or taking antihypertensive medication. Smoking was summarised as a binary variable: “yes” vs. “no”.

### Statistical analysis

Baseline characteristics were expressed as proportions, mean ± standard deviation and median (interquartile range, IQR), depending on their distribution. The main model for the primary outcome htTKV was a linear mixed model, with htTKV as outcome and coffee consumption as exposure, and adjustment for confounders (baseline age, sex, hypertension, smoking and BMI). We included a random intercept for each subject and a random slope for each subject over time in the mixed model. Most of the covariates were time-varying (coffee, hypertension, smoking and BMI) except for two which were fixed (baseline age and sex) [15]. Mixed models allowed us to track subject-specific change over time and take into account the fact that data from the same individual were not independent. p values of <0.05 were considered significant. Stata 13.1 (StataCorp, College Station, TX, USA) was used for data analysis.

## Results

### Patient characteristics

At inclusion (from April 2006 to June 2014) all 220 patients had a proven ADPKD diagnosis. After exclusion of 69 patients who did not make a minimum of 2 visits, the remaining 151 ADPKD patients, with 687 observations, were included in the analyses. At baseline, 101 (67%) patients were coffee drinkers and 50 (33%) did not drink coffee. The overall mean ± SD age was 32.8 ± 8.9 years, and 60 (40%) were female. Non-coffee drinkers compared to coffee drinkers (Table 1) were younger (28 ± 8 vs. 35 ± 8 years), had smaller kidneys at baseline (753 vs. 1118 cm^3^), and better renal function (eGFR 95.8 ± 19.5 vs. 88.2 ± 19.1 ml/min/1.73 m^2^). The majority of patients suffered from hypertension: 26 (52%) of the non-coffee drinkers and 69 (68%) of the coffee drinkers.

**Table 1:** Demographic, clinical, and laboratory data at enrolment (baseline) according to coffee consumption group

Characteristic	Total n = 151	Non-coffee drinkers n = 50	Coffee drinkers n = 101
Age, years	32.8 ± 9	27.92 ± 8	35.2 ± 8
Sex, n (%)			
Female	60 (40)	23 (46)	37(37)
Male	91 (60)	27 (54)	64 (63)
BMI, kg/m^2^ (mean ± SD) (missing = 9)	24.04 ± 4	23.62 ± 4	25.38 ± 4
eGFR, ml/min/1.73 m^2^ (missing = 2)			
Mean ± SD	90.78 ± 19	95.8 ± 19	88.23 ± 19
Median (IQR)	89.24 (78–104)	97.01 (82–113)	87.04 (75–102)
Smoking, n (%)	53 (36)	16 (33)	37 (37)
TKV, cm^3^			
Mean ± SD	1050.8 ± 685	915.8 ± 632	1117.8 ± 703
Median (IQR)	894.51 (576–1306)	752.56 (492–1038)	976.43 (603–1372)
htTKV, cm^3^/m			
Mean ± SD	595.9 ± 382	519.3 ± 364	633.9 ± 87
Median (IQR)	504.56 (333–732)	421.7 (268–613)	549.03 (353–762)
Hypertension, n (%)	95 (63)	26 (52)	69 (68)
Blood pressure, mmHg (missing = 2)			
Systolic (mean ± SD)	138.4 ± 14	136.5 ± 13	139.4 ± 15
Diastolic (mean ± SD)	89 ± 10	86.2 ± 9	90.5 ± 11
Antihypertensive drugs, n (%)	107 (71)	32 (64)	75 (74)

### Association of coffee consumption with kidney progression

**Fig. 1:**
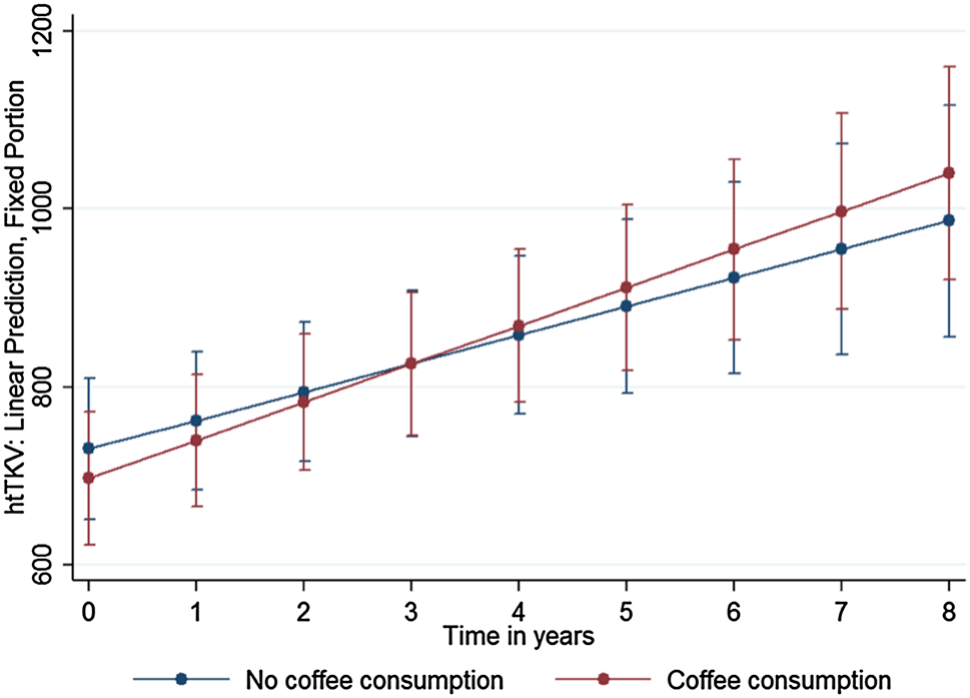
Adjusted prediction of kidney size (height adjusted total kidney volume, htTKV) with 95% confidence interval (CI), by coffee consumption group

We found in the adjusted mixed model for htTKV a smaller estimated kidney size among coffee drinkers than non-coffee drinkers [(beta)_Coffee_ = −33.13; 95% confidence interval (CI) from −72.52 to 6.34; p = 0.10] but the difference was not significant. The interaction between coffee and time in years was also not statistically significant [(beta)_Coffee*Visityr_ = 10.85; 95% CI from −1.89 to 23.58; p = 0.10], indicating only weak evidence for a steeper increase of htTKV in coffee drinkers over time. The time variable was significant in all analyses [(beta)_Visityr_ = 49.32; 95% CI from 35.49 to 63.10; p < 0.01]. Figure 1 illustrates the development of htTKV over time from the mixed model taking into account an interaction between time (in years) and coffee. The lower baseline values of coffee drinkers as well as the steeper slope over time are clearly visible.

A sensitivity analysis included the alternative time variable “number of visits since enrolment” instead of time in years and “age” as a time-varying covariate (instead of age at baseline) and showed similar results (Table 2) to the main model. A repetition of the analysis for patients with more than 2 visits (i.e. subjects were omitted if they had made <3 visits) confirmed our results. An additional sensitivity analysis without imputation of missing values for coffee showed similar results and confirmed the main analysis (Supplementary data Table 1).

**Table 2:** Association of coffee consumption with adjusted kidney size (height adjusted total kidney volume, htTKV) over time (n = 148)

Main analysis: htTKV with visit in years and baseline age			
Name	Coefficient	p	95% confidence interval (CI)
Fixed effects			
Intercept	222.19	0.14	From −76.86 to 521.25
Coffee	−33.13	0.10	From −72.52 to 6.34
Visityr	49.32	<0.01	From 35.49 to 63.10
CoffeeVisityr	10.85	0.10	From −1.89 to 23.58
Sex	−204.56	<0.01	From −316.18 to −91.19
Age at baseline	17.30	<0.01	From 11.25 to 23.67
BMI	4.78	0.08	From −0.57 to 10.14
Hypertension	−13.01	0.27	From −36.55 to 10.42
Smoker	−6.27	0.59	From −29.27 to 16.72

Group		Name	Variance
Random effect			
Patno	Intercept	333.75	19.72
Visityr		59.05	3.93
Residual		47.43	1.77

Sensitivity analysis: htTKV with number of visits and age (time-dependent)			
Name	Coefficient	p	95% CI
Fixed effects			
Intercept	−2.67	0.99	From −296.45 to 291.10
Coffee	−66.63	0.02	From −123.85 to −9.42
Visits (n)	12.67	0.01	From 2.73 to 22.61
Coffeevisit	10.17	0.03	From 1.07 to 19.26
Sex	−189.02	<0.01	From −297.85 to −80.20
Age	24.60	<0.01	From 18.93 to 30.27
BMI	3.41	0.28	From −2.78 to 9.60
Hypertension	−13.95	0.33	From −42.06 to 14.17
Smoker	−7.32	0.61	From −35.17 to 20.54

Group		Name	Variance
Random effect			
Patno	Intercept	318.12	19.90
Visits (n)		36.00	2.42
Residual		57.43	2.16

### Association of coffee consumption with eGFR

**Fig. 2:**
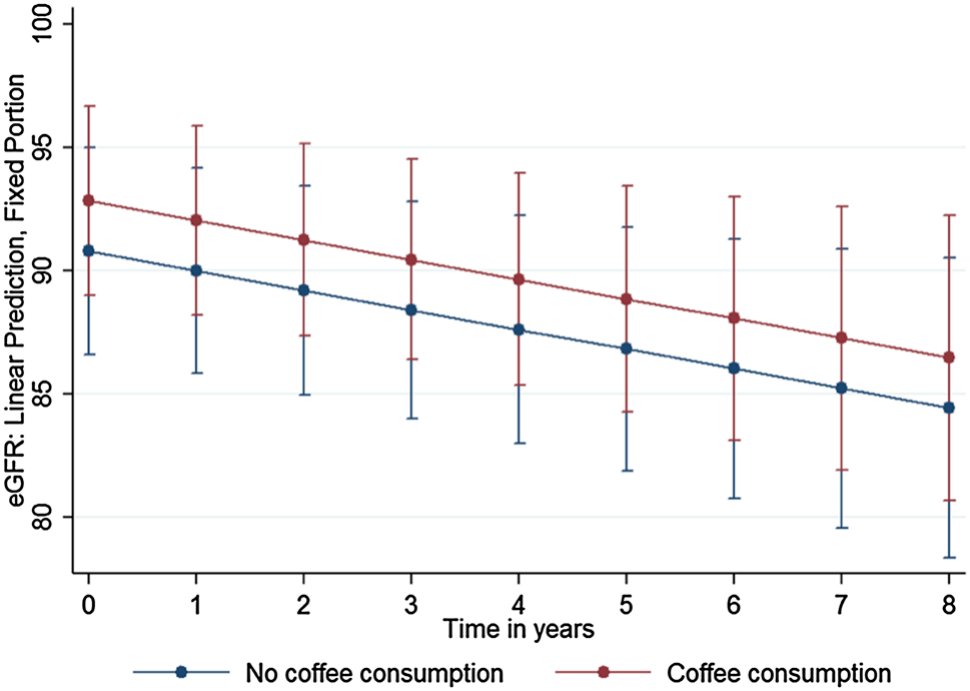
Adjusted prediction of kidney function (estimated glomerular filtration rate, eGFR) with 95% CI, by coffee consumption group

The main mixed model for eGFR was adjusted for the same confounders as the model for htTKV (Table 3). We can see that eGFR was higher among coffee than non-coffee drinkers 2 ml/min/1.73 m^2^ [(beta)_Coffee_ = 2.03; 95% −0.31 to 4.38, p = 0.089]. However, this effect was again not significant. Figure 2 illustrates our mixed model’s adjusted predictions of eGFR with 95% CI.

**Table 3:** Adjusted association of coffee consumption with kidney function (estimated glomerular filtration rate, eGFR) over time (n = 148)

Main analysis: eGFR with visits in years and baseline age			
Name	Coefficient	p	95% CI
Fixed effects			
Intercept	140.67	<0.01	From 124.38 to 156.96
Coffee	2.03	0.089	From −0.31 to 4.38
Visityr	−2.13	<0.01	From −2.67 to −1.59
Sex	0.48	0.854	From −4.69 to 5.67
Age at baseline	−1.34	<0.01	From −1.62 to −1.05
BMI	−0.17	0.418	From −0.58 to 0.24
Hypertension	−3.56	<0.01	From −5.98 to −1.15
Smoker	−1.81	0.141	From −4.22 to 0.59

Group		Name	Variance
Random effect			
Patno	Intercept	14.35	0.92
Visityr		2.44	0.23
Residual		6.21	0.22

Sensitivity analysis: eGFR with visit number and age (time-dependent)			
Name	Coefficient	p value	95% CI
Fixed effects			
Intercept	142.86	<0.01	From 126.92 to 158.79
Coffee	2.13	0.079	From −0.24 to 4.52
Visits (n)	−0.46	<0.01	From −0.83 to −0.08
Sex	0.08	0.975	From −5.04 to 5.21
Age	−1.37	<0.01	From −1.64 to −1.09
BMI	−0.18	0.385	From −0.59 to 0.23
Hypertension	−3.35	<0.01	From −5.78 to −0.92
Smoker	−1.51	0.223	From −3.93 to −0.92

Group		Name	Variance
Random effect			
Patno	Intercept	13.97	0.93
Visits (n)		1.56	0.14
Residual		6.22	0.22

The sensitivity analysis with the time variable ‘number of visits since enrolment’ and adjusted for age as a time-varying covariate (instead of age at baseline) showed similar results to the main analysis. As in the former section, the analysis for subjects with more than 2 visits (subjects were omitted if <3 visits were made) showed very similar results. An additional sensitivity analysis without replacing missing values for coffee confirmed the results of the main analysis (Supplementary data Table 2)

## Discussion

In our prospective longitudinal study of an ADPKD cohort at an early stage of disease, we did not find a statistically significant association between coffee consumption and disease progression as measured by kidney volume and function. The main analyses were corroborated by the sensitivity analyses, which assessed if results changed by including predictors for ADPKD progression as time-varying covariates. The examination of the relationships between htTKV and age as a time-varying covariate showed similar results for the slope whereas the interception point of the trajectory lines was higher (approximately for 4 visits). Our findings indicate that drinking coffee is unlikely to be a risk factor for disease progression in ADPKD patients.

One may speculate if coffee consumption even has some protective effect. The point estimates for kidney volume and function favour the coffee consumption group although not significantly so. The effect might change over time, as Figs. 1 and 2 show. The protective effect on kidney volume may diminish over time, whereas the effect of coffee consumption on kidney function was constant over time.

In fact, cohort studies in human beings have well investigated the effect of coffee consumption on various outcomes and repeatedly showed a beneficial effect. A community-based study from Hsu et al. investigated risk factors for CKD including coffee consumption. After adjustment, coffee consumption was associated with a lower risk of CKD [16]. Three large cohort studies published in 2014 investigated the association between coffee consumption and the incidence of kidney stones. Drinking coffee was independently associated with a lower risk of incident kidney stones [17]. Coffee consists of more than caffeine, it is also rich in antioxidants, to which many of its health benefits have been attributed [18]. Previous studies reported that coffee can have a positive effect on health and is a protective factor against diabetes, cancer (skin, breast, neck and head), stress, Parkinson’s disease and also heart disease [8–10]. Besides these observations, other studies have also reported negative effects of coffee consumption on blood pressure, cholesterol, serum lipid levels, and insulin resistance [19–22]. A dose-response meta-analysis of 21 longitudinal studies with nearly 1,000,000 subjects showed a non-linear association between coffee and mortality from all causes, cardiovascular disease and cancer. The highest risk reductions were observed by drinking four cups per day for all-cause mortality and three cups per day for cardiovascular mortality. Drinking coffee was not associated with increased mortality due to cancer [7]. Therefore, transferring the strong evidence of a protective effect of moderate coffee consumption vis-à-vis other diseases may imply that drinking coffee could also be beneficial for ADPKD patients in general, through its antioxidant and anti-inflammatory effects. Indeed, our results question the general recommendation to avoid drinking coffee for patients affected by ADPKD.

Currently, tolvaptan is available as a disease modifying therapy in Japan, Canada, the United States and also in Europe, where it has recently been approved [23]. Before, only co-morbidities could be treated. Besides this new medical treatment, lifestyle factors are particularly important for ADPKD patients to reduce risk factors and strengthen resources that can help prolong and stabilize renal function until end-stage renal disease (ESRD). Therefore, avoiding potential risk factors in lifestyle has been attributed an important role to delay the disease progression in patients with ADPKD.

Our results have to be interpreted in the context of the study design and setting. First, one limitation is the measurement of coffee consumption as the only source of caffeine (e.g. no soda, black tea or energy drinks were evaluated). Although it is difficult to capture coffee consumption differently, self-reported coffee consumption may be prone to misclassification, which may result in biased results. A limitation of our study is that self-reported coffee consumption does not include other sources of caffeine like cola, energy drinks and black tea. Second, the number of follow-up visits may still be too low and we may have missed important long-term effects. Moreover, our subjects did not routinely undergo genetic testing during the study visits. However, based on the mean annual kidney growth rate of 9.43% and the median kidney size at baseline of 894.51 cm^3^, it is very likely that the vast majority of our patients had PKD1 mutations.

Strengths of the presented study include its longitudinal design, the comprehensive statistical approach, the careful measurement of kidney volume and function and the well-described cohort of untreated ADPKD patients at an early disease stage.

We believe that our results are a major step forward to elucidate the role of coffee consumption in ADPKD patients. The current evidence in patients with ADPKD including this longitudinal study does not suggest an association between coffee consumption and disease progression. It may be too early to frame a recommendation for coffee consumption but it is time to at least lift the current recommendation to ‘strictly avoid’ coffee consumption. In order to come up with stronger and evidence-based recommendations, additional studies are needed to strengthen the causal inference between coffee consumption and disease progression. Additional prospective cohort studies would show how consistent the results are across studies, if there is a dose–response relationship, if there are subgroups of ADPKD patients who have different effects of coffee consumption, and if longer follow-up reveals a protective effect of coffee consumption.

In conclusion, this is the first prospective longitudinal study to investigate the long-term effect of coffee consumption in an ADPKD population, carefully controlled for confounding. Our results suggest that drinking coffee is not a risk factor for ADPKD progression and question the current recommendations against coffee consumption.

## Electronic supplementary material

Below is the link to the electronic supplementary material.

Supplementary material 1 (DOCX 22 KB)
