# Supplementary material for: Long-term effect of coffee consumption on autosomal dominant polycystic kidneys disease progression: results from the Suisse ADPKD, a Prospective Longitudinal Cohort Study
Source: J Nephrol. 2017 Apr 6;31(1):87–94. doi: 10.1007/s40620-017-0396-8 (PMC5778163; doi:10.1007/s40620-017-0396-8)
Supplement: Supplementary file 1 — Supplementary material 1 (DOCX 22 KB) [file 40620_2017_396_MOESM1_ESM.docx]

**Supplementary data table 1.** Sensitivity analysis without imputation for missing values for coffee consumption: association of coffee consumption with adjusted kidney size (height adjusted total kidney volume (htTKV)) over time (N=146 with 477 observations)

|  |  |  |  |  | |  |  |
| --- | --- | --- | --- | --- | --- | --- | --- |
| **Sensitivity Analysis: htTKV with visit in years and baseline age** | | | | | | | |
| **Fixed effects** |  |  |  |  |  |  |  |
| **Name** | **Coefficient** | **p-Value** | **95% -CI** |  |  |  |  |
| (Intercept) | 137.33 | 0.40 | from -187.84 to 462.49 | |  |  |  |
| Coffee | -44.79 | 0.07 | from -93.72 to 4.14 | |  |  |  |
| Visityr | 49.31 | <0.01 | from 33.77 to 64.85 | |  |  |  |
| CoffeeVisityr | 13.22 | 0.08 | from -2.02 to 28.45 | |  |  |  |
| Sex | -171.75 | <0.01 | from -283.19 to -60.32 | |  |  |  |
| Age Baseline | 15.41 | <0.01 | from 9.31 to 21.52 | |  |  |  |
| BMI | 8.74 | 0.02 | from 1.21 to 16.26 | |  |  |  |
| Hypertension | -4.14 | 0.83 | from -42.91 to 34.62 | |  |  |  |
| Smoke | -6.59 | 0.65 | from -35.72 to 22.53 | |  |  |  |
| **Random Effect** |  |  |  | |  |  |  |
| Group |  | Name | Variance | |  |  |  |
| Patno | (Intercept) | 318.17 | 19.47 | |  |  |  |
| Visityr |  | 60.51 | 4.39 | |  |  |  |
| Residual |  | 56.39 | 2.62 | |  |  |  |
|  |  |  |  |  | |  |  |
|  |  |  |  |  | |  |  |

**Supplementary data table 2.** Sensitivity analysis without imputation for missing values for coffee consumption: Adjusted association of coffee consumption with kidney function (estimated glomerular filtration rate ml/min/1.73 m^2^ (eGFR)) over time (N=146 with 506 observations)

|  |  |  |  |  | |  |  |
| --- | --- | --- | --- | --- | --- | --- | --- |
| **Sensitivity Analysis: eGFR with visit in years and baseline age** | | | | | | | |
| **Fixed effects** |  |  |  |  | |  |  |
| Name | Coefficient | p-Value | 95% -CI |  |  |  |  |
| (Intercept) | 140.67 | <0.01 | from 123.79 to 157.54 | |  |  |  |
| Coffee | 2.12 | 0.10 | from -0.41 to 4.65 | |  |  |  |
| Visityr | -2.01 | <0.01 | from -2.60 to -1.43 | |  |  |  |
| Sex | -0.28 | 0.91 | from -5.54 to 4.97 | |  |  |  |
| Age Baseline | -1.23 | <0.01 | from -1.51 to -0.94 | |  |  |  |
| BMI | -0.29 | 0.20 | from -0.74 to 0.15 | |  |  |  |
| Hypertension | -2.66 | 0.08 | from -5.72 to 0.39 | |  |  |  |
| Smoke | -2.43 | 0.06 | from -4.98 to 0.10 | |  |  |  |
| **Random Effect** |  |  |  | |  |  |  |
| Group |  | Name | Variance | |  |  |  |
| Patno | (Intercept) | 13.87 | 0.94 | |  |  |  |
| Visityr |  | 2.59 | 0.25 | |  |  |  |
| Residual |  | 5.97 | 0.26 | |  |  |  |
|  |  |  |  |  | |  |  |
|  |  |  |  |  | |  |  |
